# Perceptions and experiences of adult caregivers receiving mindfulness-based behavioural education: A qualitative study

**DOI:** 10.51866/oa.573

**Published:** 2024-04-17

**Authors:** Pantip Sangprasert, Pasitta Ondee, Srimuang Palungrit

**Affiliations:** 1 Ph.D., M.Sc., B.Sc., Public Health Division of Community Medicine and Family Medicine, Faculty of Medicine, Thammasat University, Pathum Thani, Thailand. Email: srimuangpa@gmail.com, srimuang@tu.ac.th; 2 RN., Ph.D., M.Ed., B.Sc., Department of Mental Health and Psychiatric Nursing, Faculty of Nursing, Mahidol University, Bangkok, Thailand.; 3 RN., Ph.D. ,M.N.S, B.Sc., A.P.N., Department of Community Health Nursing, Faculty of Nursing, Thammasat University, Pathum Thani, Thailand.

**Keywords:** Adult caregivers, Behavioural change, Mindfulness-based education, Self-efficacy, Communication

## Abstract

**Introduction:**

Adult caregivers (ACGs) are largely responsible for caring for their family members, which can increase their stress levels. This increased stress makes them more susceptible to chronic illnesses. The adoption of health-promoting behaviours, particularly through mindfulness-based behavioural education (MBBE), can significantly impact the daily habits of ACGs. However, there is limited research on this topic in the context of Thailand. Thus, this study aimed to explore the perceptions and experiences of ACGs receiving MBBE, focusing on physical, mental, social and other influential factors.

**Methods:**

A qualitative study was conducted among 19 ACGs living in the Bangkok Metropolitan Area. Focus group discussions (FGDs) and observations were conducted. The FGDs were digitally recorded, and their contents were analysed.

**Results:**

The ACGs were found to perceive and experience improvements in their mental stability and habitual behaviour. These improvements manifested as increased calmness, relaxation, clear communication, empathy, reduced risky behaviour, decreased desire for excessive consumption and travel and reduced pain. Furthermore, mindful behaviour was influenced by both internal and external personal factors as well as the specific situational environment.

**Conclusion:**

MBBE could lead to an increase in promotional behaviour, especially when combined with awareness, self-recollection and a self-efficacy approach. This finding encourages health personnel to consider incorporating regular skill practice as a complement to health education.

## Introduction

Currently, the responsibility of caring for family members falls on adults in the human development life cycle.^1^ This responsibility includes taking care of children, teenagers, spouses and ageing parents.^2^ The problems faced by older individuals are increasing globally, including in Thailand.^3,4^ In particular, health problems necessitate the presence of daily caregivers for older adults. Adult caregivers (ACGs) often face physical problems, such as chronic illness, and mental challenges, such as stress and anxiety. Additionally, they exhibit reduced social interaction within society. Notably, most family caregivers are women, and they often experience heightened stress.^5^ Numerous studies have shown that caregivers are at risk of experiencing negative psychological, physical and social health outcomes.^6^ These outcomes include chronic illness, strain, reduced social support when caring for patients with dementia^7^ and burden when caring for ageing parents with chronic illnesses.^2^ Further, research has found an elevated risk of acute myocardial infarction among young ACGs.^8^

A previous study examined the impact of caregiver burden on various aspects of individuals’ lives, including their psychological well-being, socioeconomic status, cognitive abilities and daily behavioural patterns.^9^ The findings showed that this burden can potentially cause poorer self-control regarding health and daily behavioural patterns. While many individuals are aware of the behaviours that can positively or negatively impact their health, they may struggle to control these habits. For instance, people may continue to indulge in excessive amounts of unhealthy foods due to their preference for sweet, oily, salty or fatty options. Additionally, stress and anxiety may arise from excessive worrying, which can further complicate their well-being. In the field of public health, evidence-based research has shown that risky behaviours contribute to the development of chronic physical and mental illnesses globally.^10,11^

Health education strategies (HESs) play a crucial role in modifying behaviours in certain situations. One effective approach is to raise awareness about mindfulness and its impact on cognitive perception, which can lead to a shift from risky habits to healthier behaviours.^12^ Several studies have shown that mindfulness-based interventions are more effective in reducing harmful health behaviours,^13^ promoting self-control and behavioural change related to chronic diseases^14^ and enhancing both physical and mental health outcomes.^15^ Evidence also shows that mindfulness contributes to many health-related outcomes and may promote health-related behaviours by enhancing self-regulation in clear thinking and conjunction with mental processes^16^ to improve the perceived self-efficacy (SE) of behaviours.^17^ Most mindfulness research has focused on the clinical psychological conditions of caregivers rather than on the adjustment of behaviours to prevent illness and the promotion of healthy habits. Mindfulness-based behavioural education (MBBE) contributes to the acquisition of skills and facilitates cognitive, affective and psychomotor learning processes, as outlined in Bloom’s taxonomy.^18^ However, there has been a lack of research on the impact of this intervention on the experiences of ACGs in Thailand. Previous reports recommend that individuals aged 35 years and above undergo annual physical and mental health screenings as part of the country’s chronic disease prevention plan, as outlined by the Ministry of Public Health.^19^ Therefore, there is a strong interest in utilising MBBE, along with other essential HESs, to promote healthy behaviours among this age group. Accordingly, this study aimed to explore the perceptions and experiences of ACGs who had received MBBE, focusing on various factors.

## Methods

### Study design andparticipants

A qualitative study was conducted among ACGs via focus group discussions (FGDs) and observations. Participants were purposefully selected from those who had previously attended MBBE training. The 6-week MBBE training constituted the following: Week 1: Practise mindful breathing and movement; Week 2: Practise mindful eating; Week 3: Practise mental mindfulness; Week 4: Practise mindful conversation; Week 5: Practise physical mindfulness; and Week 6: Practise holistic mindfulness every day to raise awareness, acceptance and understanding of common experiences, inspire relaxation and compassion for others on a daily basis and promote behavioural modification. The inclusion criteria were as follows: 1) age of 35–59 years, 2) main caregiver role in the family for older adults and other family members, 3) the ability to communicate in Thai and 4) absence of a history of brain conditions or psychiatric symptoms. Ultimately, a total of 19 participants were included.

### Study tool

During the FGDs, guideline questions developed by the researchers were used. These questions involved three aspects of mindful behaviour: physical, mental and social. The factors influencing mindful behaviour were evaluated based on the objectives of the research.^20^ Open-ended techniques were used to encourage free responses regarding the awareness of daily behaviours and the factors influencing them. The inquiries were reviewed for their objectiveness, consistency, clarity and language appropriacy by three experts.

The Thai notes underwent verification by three investigative experts^21^ including a physician specialising in mindfulness, a linguist and a nurse practitioner with qualitative expertise. The notes were further enhanced based on the recommendations of the experts. Subsequently, the findings were accurately translated into English.

### Data collection

Data were collected during week 8 after the 6-week MBBE training. Participants were invited to the health promotion nursing centre. The FGDs explored participant experiences. Participants were divided into three groups based on the criterion that the groups had similar characteristics. However, within each group, participants had different characteristics, including sex, age and educational level. The process was conducted by two researchers and an external individual, all of whom completed a course in qualitative research. The moderator shared clear objectives and asked questions and sub-questions for each group. Participants answered each question repeatedly until saturation, taking approximately 90–120 min. Trustworthiness was ensured. In each group, data were collected via tape recording and observation, transcribed word by word and merged. External validity was examined using triangulation by the three experts.

### Data analysis

A five-step content analysis was conducted, which included data organisation, information coding, data clustering, data interpretation and conclusion.^22^ Based on the research objectives, information coding included awareness, acceptance and self-recollection, with descriptions of emotions and sensations by automatic memory of daily events. Physically mindful behaviours were evaluated through individual feelings and bodily sensations, including breathing in all daily activities; mentally mindful behaviours through individual emotions in daily activities; socially mindful behaviours through personal understanding of thoughts and emotions while interacting with others; and factors influencing mindful behaviours through remembered events contextually affecting physical and psychological moods.

## Results

**Figure 1:**
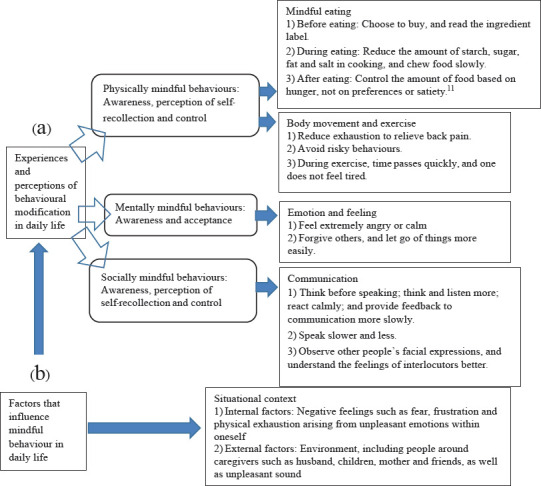
Summary of the focus group discussion for exploring the experiences and perceptions of adult caregivers in caring for older people and other family members.

Figure 1 summarises the FGD for exploring the experiences of the participants based on three aspects: physical (body movement and food consumption), mental (emotions and feelings) and social (communication). The determinants of behaviours included internal and external factors relative to the situational context. MBBE activities encompassed group processes, and the perceptions of the participants were analysed and used to evaluate changes in their daily experiences.

### Participant demographics

A total of 19 ACGs participated in this study. Their age ranged from 35 to 59 years (mean: 52.68 years). Most participants were women (89.47%); almost half were unmarried; and 31.57% had non-communicable chronic diseases, including hypertension, diabetes mellitus, dyslipidaemia and cancer.

### Daily behavioural modification experience

Figure 1a presents the sharing of self-contained internal experiences in daily behavioural modification. Physically mindful behaviours were divided into mindful eating and body movement/exercise.

Mindful eating was associated with different experiences before and after mindfulness practice. The participants shared consumption of unhealthy foods and experiences with mindful ingestion, with food ingredients and emotional consumption tested. Some participants stated that they preselected healthy foods by reading labels and avoiding starch, sugar and salt. Others chewed slower and dieted until they lost weight to control diabetes mellitus. Mindful eating was divided into three phases: 1) before (buying, selecting and reading ingredient labels); 2) during (reducing starch, sugar, fat and salt in cooking and slowly chewing food); and 3) after (controlling the portions of food not based on preferences or satiety). Two female participants shared:

*‘Mindfulness changed to newly behaviour in several participants on aware of ingredients when buying ready-to-eat food. They select and read ingredient labels for sodium and fat levels, with diabetics noting sugar contents... I chew food slower, cook for myself with modest portions and avoid ready-made meals. I consume less starch, sugar, fat and salt. I was diagnosed with diabetes but am no longer required to take medication. (woman, 57 years old)*

*‘Mindfulness leads to more concentration, intelligence and awareness. As a diabetic, I can control my diet’. (woman, 35years old)*

Mindful body movement and exercise were associated with perceived physical gestures at present. Breathing is a bodily function, and being conscious of it signifies mindfulness of the present moment. Herein, the participants reported that breathing awareness reduced exercise-related fatigue, exhaustion and back pain. While also avoiding physical risks when riding vehicles, some participants eliminated risky actions, such as jumping on buses or accelerating motorcycles. Two participants commented:

*‘My back pain was relieved by mindful breathing. Previously, I never thought about my breath’. (man, 49 years old)*

*I remind myself not to jump on buses and not to ride motorcycles fast. I am more careful and calmer. Previously, I rode at 60–80 kilometres per hour. Now, I ride at 40–50 kilometres per hour in the slow lane. I wait for other cars to pass. Before, if someone overtook me, I would be angered. . Before, when I walked up the footbridge, I was tired. Now, I’m not tired because I focus on inhaling and exhaling when doing typical activities’. (woman, 55 years old)*

Mentally mindful behaviours involved the awareness and acceptance of emotions and feelings in different daily situations. Some participants noticed changes in their current thoughts and emotions, such as feeling angry when someone jumps a queue or being distracted during prayer. The participants experienced less irritability and greater calmness and forgiveness. Two participants shared:

*I would be angered if someone jumped a queue, but now, I am calmer’. (woman, 35years old)*

*‘Previously, I prayed five times daily but lacked concentration. Now, mindful breathing calms me down. (man, 49years old)*

Socially mindful behaviours involved thoughts and emotions related to communication. Some participants admitted frustration when talking to older people who are unable to hear them properly. Mindful breathing made them more considerate, causing them to speak more slowly and less frequently with greater tranquillity. The participants reported thinking before speaking, listening more, reacting more calmly and slowly and understanding the feelings of interlocutors better based on their facial expressions when communicating. Two participants said:

*I can suppress frustration when talking to the older persons who dont hear me despite sitting nearby. I concentrate on inhaling to speak slower, and they can hear me more clearly’. (man, 49 years old)*

*I think and listen more and speak slower and less. In the past, I responded to my husband immediately during arguments. Now, I react more calmly’. (woman, 55 years old)*

### Factors influencing daily mindful behaviours

Figure 1b presents the sharing of factors influencing mindful behaviours in daily life.

Mindful breathing patterns influenced the participants’ inability to control their thoughts, emotions and actions. Their mindful behaviours were affected by the following situational contexts: 1) internal factors, including negative feelings such as fear, frustration and physical exhaustion arising from unpleasant emotions within oneself, and 2) external factors, such as the environment including family members and friends as well as unpleasant sound from pets. Three female participants commented:

*‘Anger can cause me to lose my train of thought, like fear’. (woman, 35 years old)*

*‘Much housework and physical weakness prevent me from practising mindfulness or exercising. (woman, 59 years old)*

*I care for my mother, who is bedridden and suffers from diabetes, hypertension and dyslipidaemia. She is worried aboutfamily conflict, so I mindfully tell her that all of us have individual karma. (woman, 55 years old)*

## Discussion

In this study, the ACGs demonstrated an enhanced level of focus, mindfulness and positive mindset as a result of practising self-management and self-control. This improvement was evident through their ability to adapt both cognitively and behaviourally. Additionally, some ACGs exhibited a heightened sense of empathy.^16^ The intervention contributed to the acquisition of skills and facilitated cognitive, affective and psychomotor learning processes, as outlined in Bloom’s taxonomy, 18 which were reinforced through training based on Dale’s cone of experience.^23^ In general, interventions facilitate a clear understanding of the body’s movements and the mind’s thoughts in the present moment.^24^

In this study, the occurrence of the terms ‘previous’ and ‘current’ was observed in certain statements, indicating a mixed emotional perception. The FGDs on the perceptions and experiences of the ACGs revealed the following findings: First, physically mindful behaviours, in contrast to conventional health education approaches that emphasise specificity, involved actively engaging in physical body movements or exercises while carefully observing and articulating the subsequent impact on the respiratory function, heart rate, muscle strength and emotional state. Some ACGs experienced pain relief or corporeal fatigue, which aligns with a previous report^25^ on the efficacy of a mindfulness-based stress reduction technique in reducing the perceived chronic headache as well as a report^26^ on the effectiveness in managing chronic pain. When the ACGs practised mindful breathing while eating and exercising, their appetites diminished, and their weight decreased. The aforementioned events demonstrated the advantages of consumption by promoting awareness and acceptance, aligning with the notion of eating for sustenance rather than for indulgence. This finding showcased the effectiveness of systematic reviews in the field of mindfulness research, demonstrating the efficacy of mindfulness interventions in reducing emotional eating tendencies and facilitating weight loss.^27^

Second, mentally mindful behaviours were intrinsically internal, thus making them challenging to observe. When the ACGs demonstrated improved self-control with energised and relaxed moods by mindful breathing, their psychological states reflected emotional and behavioural flexibilities.^28^ Selfrecollection has the potential to enhance selfcontrol across six interconnected channels of perception: visual, auditory, olfactory, gustatory, tactile and emotional. Some individuals exhibit a more rapid mastery of their emotions in irritating situations than do those who remain calm. Additionally, the practice of mindfulness has been found to reduce negative thoughts and promote a neutral emotional state. This finding aligns with a previous report on the relationship between mindfulness and the promotion of positive emotions and behavioural change for improved health.^6^

Third, the ACGs demonstrated socially mindful behaviours after engaging in mindful conversations. While speaking, they focused on their breathing, and while listening, they paid attention to their inhalation and exhalation. The deliberate and attentive approach of mindful communication facilitated a clear comprehension and understanding of the narrative. Additionally, the ACGs carefully considered their words before speaking, refrained from using negative language, showed greater understanding towards others and developed empathy. In another study, ACGs used techniques such as staying calm and providing stimulation for self-management. This approach influenced family behavioural changes and moods through distraction, resting and discussion of emotions and experiences.^29^

Finally, mindfulness was practised while focusing on the present physical and psychological states.^24^ Although the behavioural changes noted in this study are similar to the general behaviours in everyday life, repeated behaviours can lead to injury and chronic illness.^11^ Moreover, 31.57% of the ACGs in this study had hypertension, diabetes mellitus, dyslipidaemia and cancer. Internal factors impaired their daily awareness and reduced their self-acceptance of negative emotions and body stressors. These situational contexts influenced mindful behaviours, either promoting or hindering daily mindfulness in relation to exposure to external stimuli affecting emotions and body stressors.

### Implications

As ACGs recall experiences, they are likely to build mental stability and stop repeating risky habitual behaviours. This finding challenges health personnel to consider incorporating regular skill practice as a complement to health education. In addition, continuous self-training and dissemination of practices to family members can ensure the sustainability of health-promoting behaviours.

### Strengths and limitations

This study has strengths, including its focus on self-awareness and the transformation of the inner world following mindfulness training. It also serves as a platform for sharing knowledge. Conversely, the study has limitations. The data on the experiences of the participants were descriptive. Further, the study explored the experiences of the participants after the training but did not measure the effectiveness of the training itself.

## Conclusion

The FGDs and observations revealed that awareness and experience can cultivate mindfulness, leading to self-recognition, control, acceptance and SE. These qualities can be applied to promote health behavioural modifications in daily life, including the performance of physical activity (eating, movement and exercise), management of mental well-being (emotions) and improvement of social interactions (communication). Such modifications also encompass the consideration of internal and external factors, contributing to holistic health and well-being. Continuous self-training and dissemination of practices to family members can ensure the sustainability of health-promoting behaviours. Nonetheless, further studies are needed to explore the internal and external factors affecting the reduction of mindfulness.

## Data sharing statement

The data are kept by the principal investigator. Requests for access to the data can be made to the principal investigator.

**How does this paper make a difference in general practice?**

- The findings provide insights into the perceptions and experiences of adult caregivers regarding behavioural modifications through mindfulness training.
- Mindful eating helps build contemplative wisdom among adult caregivers, ultimately benefitting patients.
- Mindful communication creates clear perceptions, cultivates empathy and reduces conflicts among families, neighbours and society.
